# BigDataProcessor2: a free and open-source Fiji plugin for inspection and processing of TB sized image data

**DOI:** 10.1093/bioinformatics/btab106

**Published:** 2021-02-17

**Authors:** Christian Tischer, Ashis Ravindran, Sabine Reither, Nicolas Chiaruttini, Rainer Pepperkok, Nils Norlin

**Affiliations:** Centre for Bioimage Analysis, European Molecular Biology Laboratory (EMBL), 69117, Heidelberg, Germany; Advanced Light Microscopy Facility, EMBL, 69117, Heidelberg, Germany; Cell Biology and Biophysics Unit, EMBL, 69117, Heidelberg, Germany; University of Heidelberg, Department of Scientific Computing, 69120, Heidelberg, Germany; Advanced Light Microscopy Facility, EMBL, 69117, Heidelberg, Germany; Cell Biology and Biophysics Unit, EMBL, 69117, Heidelberg, Germany; BioImaging & Optics Platform (BIOP), Faculty of Life Sciences (SV), Ecole Polytechnique Fédérale de Lausanne, CH-1015 Lausanne, Switzerland; Advanced Light Microscopy Facility, EMBL, 69117, Heidelberg, Germany; Cell Biology and Biophysics Unit, EMBL, 69117, Heidelberg, Germany; Cell Biology and Biophysics Unit, EMBL, 69117, Heidelberg, Germany; Department of Experimental Medical Science, 221 84, Lund University, Lund, Sweden; Lund University Bioimaging Centre, 222 41, Lund University, Lund, Sweden

## Abstract

**Summary:**

Modern bioimaging and related areas such as sensor technology have undergone tremendous development over the last few years. As a result, contemporary imaging techniques, particularly electron microscopy (EM) and light sheet microscopy, can frequently generate datasets attaining sizes of several terabytes (TB). As a consequence, even seemingly simple data operations such as cropping, chromatic- and drift-corrections and even visualisation, poses challenges when applied to thousands of time points or tiles. To address this we developed BigDataProcessor2—a Fiji plugin facilitating processing workflows for TB sized image datasets.

**Availability and implementation:**

BigDataProcessor2 is available as a Fiji plugin via the BigDataProcessor update site. The application is implemented in Java and the code is publicly available on GitHub (https://github.com/bigdataprocessor/bigdataprocessor2).

**Supplementary information:**

Supplementary data are available at *Bioinformatics* online.

## 1 Introduction

Inspection and processing of TB sized image data as produced by state-of-the-art light-sheet and volume electron microscopy poses several practical challenges (Power and Huisken, 2017). Even image inspection can be burdensome, because loading the entire dataset from disk into Random Access Memory (RAM), as it is usually done for conventional MB to GB sized data, is not feasible due to the limitations of a standard computer’s RAM. In addition, pixel-wise image processing operations on the whole dataset can take hours and may require data duplication on disk as the processed images cannot be held in RAM.

The challenges of big image data inspection can be addressed by lazy-loading schemes where only the portion of the data is loaded into RAM that is needed to render the current view on the computer monitor. There are several commercial and open source solutions that adopt lazy-loading to interactively render big image data. The rendering modes include fixed angle 2D slicing: ImageJ (Schneider *et al.*, 2012); arbitrary angle 2D slicing: BigDataViewer (Pietzsch *et al.*, 2015); and 3D volume rendering: Imaris (Oxford Instruments), Arivis Vision4D (Arivis AG), Amira (Thermo Fisher Scientific), Vaa3D (Bria *et al.*, 2016) and TDat (Li *et al.*, 2017). However, except for the fixed angle 2D slicing mode, these solutions require the data to be saved in specifically chunked multi-resolution file formats that enable efficient loading of arbitrary data portions from disk into RAM. Due to write performance considerations, raw microscopy data is typically not saved in a format that is compatible with those requirements. In addition, raw microscopy data can have further shortcomings. For example, only parts of the acquired data may be of actual interest, either because larger fields of view have been acquired to compensate for unpredictable sample motion, or scientifically interesting phenomena have only occurred in specific parts of the imaged sample. Also, pixel density and bit-depth can be unnecessarily high, because camera-based microscope systems with fixed pixel size and bit-depth have been used. Moreover, there may be chromatic aberrations or other shifts between acquired data channels or time points.

Taken together, to render raw microscopy data amenable for analysis it typically needs to be re-saved in a suitable file format and a number of processing operations such as cropping, binning, bit-depth conversion, channel shift and drift correction might need to be applied. It is important to realize that, if performed sequentially, each of these processing steps requires loading, processing and re-saving of the entire initially TB sized dataset, which, taken together, could take several hours or even days. It would be much more efficient to load the raw data, apply all processing steps and then re-save the data only once.

**Fig. 1.**
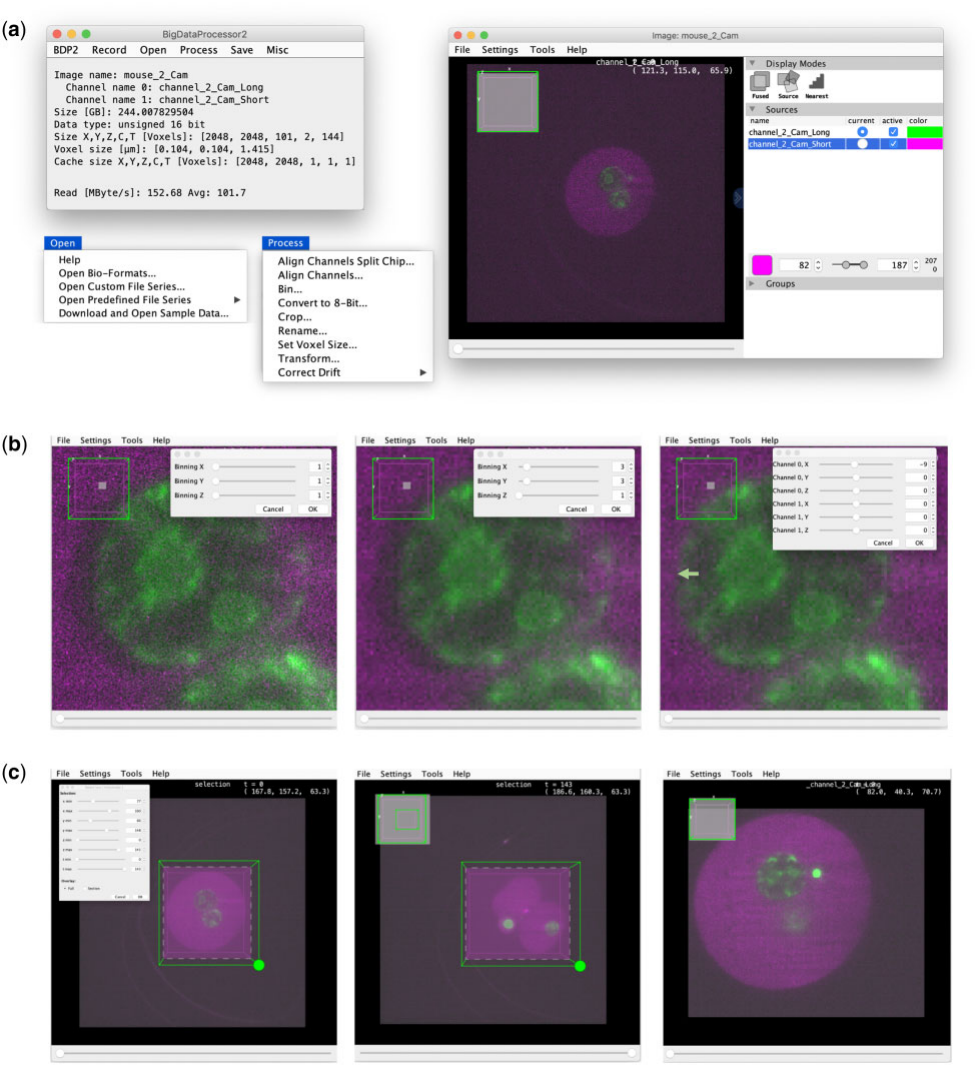
Overview of the user interface and an example workflow in BDP2. (**a**) Image browsing. Left: The main BDP2 window with the Open and Process dropdown menus expanded (see also Supplementary Note S1). Right: BigDataViewer's user interface. (**b**) Binning and channel alignment. Left: Original data (zoomed in). Middle: 3×3 binning in *X*&*Y*, reducing noise and the size of the dataset by a factor of 3×3 = 9. Binning (and all other processing operations) is performed by means of lazy computation and can thus be configured interactively even for TB sized datasets. Right: Channel alignment, correcting a shift (green arrow) of the green relative to the magenta channel. (**c**) Cropping. Left: First time point of the dataset with interactive cropping user interface. Middle: Last time point of the dataset with enlarged cropping area to include all relevant data. Right: Cropped data, showing again the first time point. Thanks to BDP2’s lazy-loading and lazy-processing the above steps (a–c) can be executed in a few minutes. Finally, the data could be re-saved using the Save menu (not shown). In this example, binning and cropping helped to reduce the size of the data from 244 to 4.8 GB without loss of biologically relevant information

To this end, making use of ImageJ’s virtual stacks (Schneider *et al.*, 2012) for lazy-loading of big image data we previously developed BigDataProcessor1 (https://github.com/bigdataprocessor/bigdataprocessor1), a Fiji plugin (Schindelin *et al.*, 2012) facilitating interactive browsing, processing and re-saving of TB sized microscopy raw data. BigDataProcessor1 has proven useful to conduct big image data processing workflows similar to the one shown in Figure 1 (e.g. Alladin *et al.*, 2020, Villani *et al.*, 2019, Wolny *et al.*, 2020). We thus decided to reimplement and enhance BigDataProcessor1 using software libraries dedicated to lazy-loading and lazy-processing of big image data (Supplementary Note S9). The resulting BigDataProcessor2 is described in this application note.

## 2 Implementation and application

BigDataProcessor2 (BDP2, https://github.com/bigdataprocessor/bigdataprocessor2) is built on ImgLib2 (Pietzsch *et al.*, 2012), a Java library for processing of n-dimensional big image data. BDP2 can be installed by activating the ‘BigDataProcessor’ Fiji update site. All functionality is accessible both via a graphical user interface (Fig. 1a) and via recordable scripts (Supplementary Note S2). BDP2 supports loading of 5D (*x*, *y*, *z*, channel and time) image data via Bio-Formats (Linkert *et al.*, 2010) as well as loading TIFF (https://www.adobe.io/open/standards/TIFF.html) and some HDF5 (The HDF Group, 1997) based image files series (Supplementary Note S3), thereby accommodating a majority of image data formats currently occurring in light sheet and volume electron microscopy. For visualisation, BDP2 employs BigDataViewer (Pietzsch *et al.*, 2015), which provides arbitrary plane slicing of 5D image data. As one microscopy camera image typically contains only a few MB of data, it fits readily into RAM and can be loaded within sub-seconds, given a data access bandwidth larger than or equal to a few MB/s. These bandwidths are nowadays typically available (Supplementary Note S7) and as such BDP2’s lazy-loading scheme allows for interactive browsing of TB sized image data on a standard computer (Supplementary Note S8).

Importantly, BDP2 also provides lazy image processing operations reducing computations to only the pixels needed to render the currently viewed image plane (Supplementary Fig. S1 and Supplementary Note S9). At present, we support the following lazy processing operations (Supplementary Note S4): affine transformed viewing, cropping, binning, bit-depth conversion, drift correction and channel alignment (chromatic shift correction, split chip acquisition). Additional processing operations can be contributed via the SciJava plugin framework (see https://github.com/bigdataprocessor/bigdataprocessor2/blob/master/CONTRIBUTE.md). The compute times of currently available operations are typically in the sub-second range for one image plane (Supplementary Note S7) such that the user can interactively configure all image processing steps while inspecting arbitrary locations in the sample (Fig. 1 and Supplementary Movies S1 and S2). Once all processing steps have been configured, the dataset can be re-saved for further analysis. We currently provide saving in TIFF and HDF5 based formats, including lossless compression, with one file per 3D volume (Supplementary Note S5). For TIFF, we also support saving each plane to a separate file. For HDF5, we save the data in a chunked pyramidal format allowing for efficient viewing with both BigDataViewer and Imaris (Imaris v9.0, Bitplane AG).

## 3 Discussion and conclusions

Visualisation and processing of an image dataset conventionally comprises loading this dataset from a storage device such as a hard disk into a computer's RAM from where it is accessed and processed by the computer's CPU. With the increasing size of image data in biological research this mode of operation is becoming challenged.

Due to the size and to ensure data integrity, datasets are typically not stored on the processing computer itself but inside the research institute's data centre, where it is accessed via a local area network (LAN) connection. Loading a medium sized light sheet dataset (16 bit, 2048×2048×100×2×100; *x*, *y*, *z*, channels, time points) via a 1 Gbit/s LAN connection would take 22 min and require 168 GB of RAM. Applying a typical processing operation such as a 3×3×1 (*x*, *y*, *z*) average binning using ImageJ’s Transform>Bin command on a 2.5 GHz Intel Core i7 (i7-7660U) on the whole dataset would take about 158 s (about 0.5 Giga voxels/s). This example shows that processing operations on whole big image datasets require high-end computer hardware with a large RAM and parameters of processing operations cannot be interactively configured as they take more than a couple of seconds to complete. Even if there were hardware solutions to achieve user-friendly real-time performance we argue it is more compute resource efficient to only process and inspect a representative subset of the data to judge whether a processing step is adequately configured.

Once an appropriate processing pipeline is configured, the whole dataset can be efficiently processed and re-saved in one go. Notably, the processed data can be significantly smaller than the raw data (Fig. 1) and thus much more efficient to work with. Unfortunately, no standardized big image data file format has yet emerged and thus one may have to re-save the data multiple times in formats optimized for specific image analysis and visualization software. Thus, we consider the establishment of a standard big image data file format as a very important endeavour for the bioimaging community within the next few years (Swedlow *et al.*, 2020).

In conclusion, we consider our BigDataProcessor2 Fiji plugin to substantially simplify the inspection and processing of big image data. As big image data is becoming increasingly prevalent we are positive that the already existing user base will grow even further in the future.

## Supplementary Material

btab106_Supplementary_Data
