# Supplementary material for: BigDataProcessor2: a free and open-source Fiji plugin for inspection and processing of TB sized image data
Source: Bioinformatics. 2021 Feb 17;37(18):3079–81. doi: 10.1093/bioinformatics/btab106 (PMC8479660; doi:10.1093/bioinformatics/btab106)
Supplement: btab106_Supplementary_Data [file btab106_supplementary_data.zip › BigDataProcessor2_3rdRevision_Supplementary Information_FINAL.pdf]

Supplementary Information

BigDataProcessor2: A free and open-source tool for inspection and processing of TB sized image data.

Christian Tischer[1-3], Ashis Ravindran[4], Sabine Reither[2,3], Nicolas Chiaruttini[5], Rainer Pepperkok[2,3] & Nils Norlin[3,6,7]

[1]Centre for Bioimage Analysis, European Molecular Biology Laboratory (EMBL), Heidelberg, Germany
[2]Advanced Light Microscopy Facility, EMBL, Heidelberg, Germany
[3]Cell Biology and Biophysics Unit, EMBL, Heidelberg, Germany
[4]University of Heidelberg, Germany
[5]BioImaging & Optics Platform (BIOP), Faculty of Life Sciences (SV), Ecole Polytechnique Fédérale de Lausanne, Switzerland
[6]Dept. of Experimental Medical Science Lund University, Sweden.
[7]Lund University Bioimaging Centre, Lund University, Sweden.

Supplementary Figure 1

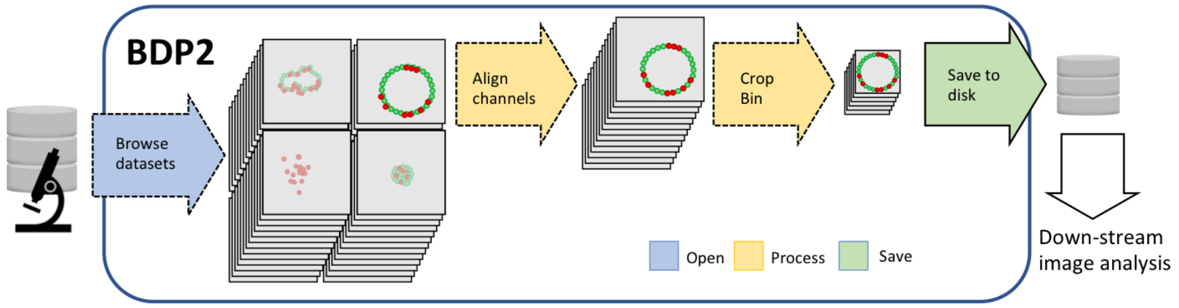

Supplementary Figure 1: Schematic representation of a lazy-processing workflow in BigDataProcessor2. Dashed arrows represent lazy-computation, where only the pixels needed to render the currently viewed image plane are loaded and processed. The complete data browsing, data selection and data processing workflow can be configured in a few minutes even for TB-sized image data. Only the final saving to disk requires processing of the whole data set and will take a correspondingly long time (up to hours).

Supplementary Note 1: Main user interface

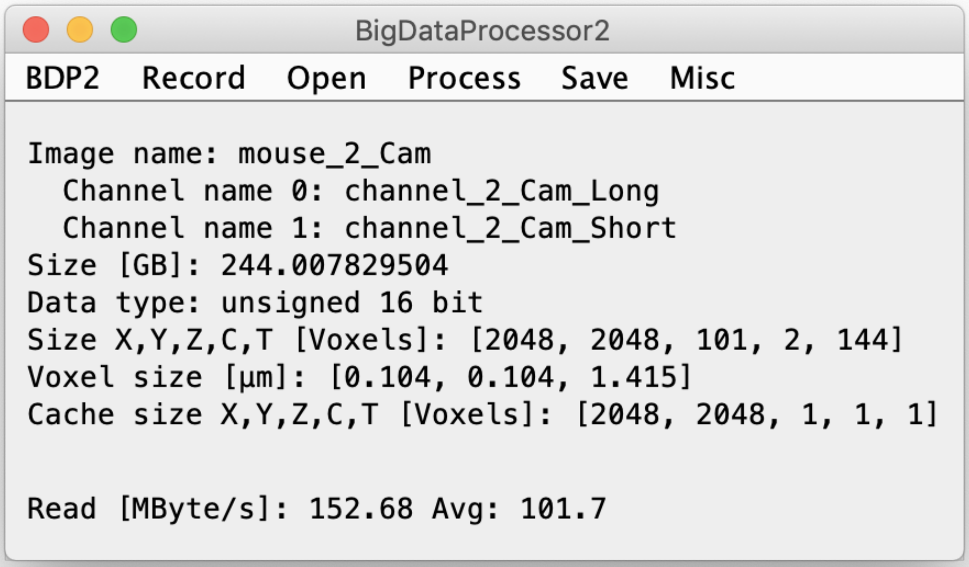

Supplementary Figure 2: The BDP2 main user interface.

BDP2 comes with its own user interface (UI) where all functionality can be accessed. The UI shows information about the currently active image as well as the current and average image data reading speed. It is possible to have multiple images (BigDataViewer windows, see Figure 1 in the main text) open at the same time. Following the usual ImageJ convention, the "active" image is the one that you clicked on last.

Supplementary Note 2: Recording

Record > Record...
Enable/ disable macro recording. The user can choose to turn macro recording on and off and also select from three recording languages, namely the IJ1 Macro language, Jython (Juneau et al. 2010) and JavaScript (https://en.wikipedia.org/wiki/JavaScript).
Motivation: Macro recording is one of ImageJ's greatest features as it allows users without programming experience to record reusable scripts. It can be used for automation but also for sharing, documentation and publishing. In our experience, next to the IJ1 Macro language, Jython is the second most popular scripting language for ImageJ, most likely due to the overall popularity of python (https://insights.stackoverflow.com/survey/2019#most-popular-technologies). We also support JavaScript due to its increasing popularity and importance in web based applications. ImageJ supports many more scripting languages, however, we decided to limit our support to a few in order to guide the user in their choice.

Example Recorded IJ1 Macro Script

```
run("BDP2 Open Luxendo Channels...", "viewingmodality=[Show in
new viewer] directory=[data] enablearbitraryplaneslicing=false
stackindex=6 channels=[channel_2_Cam_Long] ");
run("BDP2 Crop...", "inputimage=[in] outputimagename=[in-crop]
viewingmodality=[Show in new viewer] minx=307 miny=576 minz=44
minc=0 mint=0 maxx=1730 maxy=1644 maxz=54 maxc=0 maxt=9 ");
run("BDP2 Bin...", "inputimage=[in-crop] outputimagename=[in-
crop-binned] viewingmodality=[Show in current viewer]
binwidthxpixels=3 binwidthypixels=3 binwidthzpixels=1 ");
run("BDP2 Save As...", "inputimage=[in-crop-binned]
directory=[data-out] numiothreads=1 numprocessingthreads=4
filetype=[TIFFVolumes] saveprojections=true savevolumes=true
tiffcompression=[None] tstart=0 tend=9 ");
```

Example Recorded Jython Script

```
# To run this script, please select language: Python
from de.embl.cba.bdp2 import BigDataProcessor2;
import java;
from jarray import array;
from de.embl.cba.bdp2.save import SavingSettings;
from de.embl.cba.bdp2.save import SaveFileType;

# Open Luxendo HDF5 File Series...
image = BigDataProcessor2.openHDF5Series( "data/mouse_2_Cam",
".*stack_6_(?<C1>channel_.*)/(?<C2>Cam_.*)_(?<T>\d+).h5",
"Data", ["channel_2_Cam_Long"] );

# BigDataProcessor2.showImage( image, True );

# Crop...
image = BigDataProcessor2.crop( image,
[307,576,44,0,0,1730,1644,54,0,9] );
image.setName( "in-crop" );

# Bin...
image = BigDataProcessor2.bin( image, [3,3,1,1,1] );
image.setName( "in-crop-binned" );

# Save image
savingSettings = SavingSettings.getDefaults();
savingSettings.volumesFilePathStump = "data/mouse_2_Cam-crop-
binned";
savingSettings.projectionsFilePathStump = "data/mouse_2_Cam-
crop-binned";
savingSettings.numIOThreads = 1;
savingSettings.numProcessingThreads = 4;
savingSettings.fileType = SaveFileType.TIFFVolumes;
```

```
savingSettings.saveProjections = True;
savingSettings.saveVolumes = True;
savingSettings.compression = "None";
savingSettings.tStart = 0;
savingSettings.tEnd = 9;
BigDataProcessor2.saveImageAndWaitUntilDone( image,
savingSettings );
```

Example Recorded JavaScript

```
// To run this script, please select language: JavaScript
importClass(Packages.de.embl.cba.bdp2.BigDataProcessor2);
importClass(Packages.de.embl.cba.bdp2.save.SavingSettings);
importClass(Packages.de.embl.cba.bdp2.save.SaveFileType);

// Open Position And Channel Subset...
image = BigDataProcessor2.openHDF5Series( "data/mouse_2_Cam",
".*stack_6_(?<C1>channel_.*)/(?<C2>Cam_.*)_(?<T>\\d+).h5",
"Data", ["channel_2_Cam_Long","channel_2_Cam_Short"] );
// BigDataProcessor2.showImage( image, true );

// Crop...
image = BigDataProcessor2.crop( image,
[307,576,44,0,0,1730,1644,54,0,9]
 );
image.setName( "mouse_2_Cam-crop" );

// Bin...
image = BigDataProcessor2.bin( image, [3,3,1,1,1] );
image.setName( "mouse_2_Cam-crop-binned" );

// Save...
savingSettings = SavingSettings.getDefaults();
savingSettings.volumesFilePathStump = "data/mouse_2_Cam-crop-
binned";
savingSettings.projectionsFilePathStump = "data/mouse_2_Cam-
crop-binned";
savingSettings.numIOThreads = 1;
savingSettings.numProcessingThreads = 4;
savingSettings.fileType = SaveFileType.TIFFVolumes;
savingSettings.saveProjections = true;
savingSettings.saveVolumes = true;
savingSettings.compression = "None";
savingSettings.tStart = 0;
savingSettings.tEnd = 9;
BigDataProcessor2.saveImageAndWaitUntilDone( image,
savingSettings );
```

Supplementary Note 3: File Opening

Open > Help
Provides detailed information about below menu items for loading big image data. In particular, it provides a list of regular expressions that can be used to open custom image file series.

Open > Open Bio-Formats…
Uses the Bio-Formats library (Linkert et al. 2010) to read from 140+ image file formats. It tries to do so lazily and can thus also work for TB sized data, but performance may depend on the image data type. If you want to read from multi file series TIFF or HDF5 data with high performance we recommend using [Open Custom File Series…] (see below).

Open > Open Custom File Series…
Open datasets consisting of a collection of TIFF or HDF5 volumes. The assignment of each file (volume) to a channel and time point can be specified by a regular expression. Please see [Open > Help] for more detailed information. Motivation: TIFF and HDF5 are common file formats for big image data. For example, Viventis Microscopy (https://www.viventis-microscopy.com) and Luxendo (https://luxendo.eu) save their light sheet data in multi file TIFF and HDF5 series, respectively. With this menu item we support efficient lazy loading from data saved in such file formats. As determining the correct regular expression can be somewhat cumbersome, we provide convenience menu items for prevalent cases (see below [Open > Open Predefined File Series]).

Open > Open Predefined File Series > Open EM TIFF Plane File Series...
Opens a single folder with TIFF single plane files. Each file will be assigned to one z-plane in a dataset with one color and one time point. Motivation: This is a typical format for volume EM data to be stored in and we wanted to relieve users from the burden to type in the regular expression for this.

Open > Open Predefined File Series > Open Leica DSL TIFF File Series...
Opens datasets acquired with Leica DSL microscopes, choosing "Auto-Save, Data type: TIFF, Compression: Uncompressed" as an option within the Leica acquisition software. Motivation: Leica's naming scheme would require entering a complex regular expression and we thus implemented this convenience opening functionality.

Open > Open Predefined File Series > Open Luxendo HDF5 File Series...
Open datasets acquired with Luxendo (https://luxendo.eu) light sheet microscopes. Motivation: Luxendo uses an HDF5 based file format. We added convenience functionality for opening those files without the need to enter complex regular expressions.

Open > Open Predefined File Series > Open Viventis TIFF File Series...
Open datasets acquired with Viventis Microscopy (https://www.viventis-microscopy.com) light sheet microscopes. Motivation: Viventis uses a TIFF based file format. We added convenience functionality for opening those files without the need to enter regular expressions.

Open > Download and Open Sample Data...
Download and open sample data stored in the BioStudies archive (Sarkans et al. 2017). Access data directly in the archive via this link:
https://www.ebi.ac.uk/biostudies/studies/S-BSST417?query=bigdataprocessor2
Motivation: Conveniently accessible example data is useful to explore and teach BigDataProcessor2 functionality without the need to prepare suitable input data.

Supplementary Note 4: Processing

Process > Align Channels Split Chip…
Specify two crop regions in one channel and convert those regions into two channels, i.e. the number of channels of the resulting image is increased by one. Motivation: For the sake of acquisition speed, some fluorescence microscope systems acquire the signal of several fluorescence channels simultaneously on the same camera chip. Thus, we provide the functionality to convert such data into a conventional multi-channel data set by aligning the channels from a “split chip”.

Process > Align Channels…
Shift one channel in relation to the other to compensate pixel offsets e.g. due to chromatic shifts. Motivation: Chromatic shifts either due to optics being corrected only for a given wavelength range, or parallel acquisition of two channels on two cameras can lead to offsets between the two channels / images. We, therefore, provide the functionality to correct for such channel shifts in x, y and z.

Process > Bin…
Performs arbitrary binning along x, y and z coordinates. Motivation: For camera-based microscopy systems the effective pixel size often cannot be freely chosen during acquisition. Thus, the user may be forced to oversample, leading to large data volumes and potentially significantly increased image processing times. Thus, binning the data post-acquisition can be very useful as it both reduces data size (and noise), often without compromising scientific accuracy.

Process > Convert to 8-bit…
Convert the data set from 16 to 8-bit depth. Motivation: Cameras typically produce image data at 12, 14, or 16 bit-depths. For many image analysis tasks, 8-bit depth is sufficient allowing the user to reduce data size by a factor of 2. However, converting 16-bit to 8-bit data is not trivial as it entails deciding on a specific mapping from the higher to the lower bit-depth, which will lose information. Choosing a mapping of 0-65535 to 0-255 can lead to a low dynamic range in the 8-bit range especially when the input contains only a subset of the full 16-bit range. Also mapping max(image) to 255 and min(image) to 0 can be sub-optimal if there are spurious pixels with very high values, again leading to a low dynamic range for the relevant grey values in the 8-bit converted data. We thus provide the possibility to interactively specify a mapping while browsing the data set to inspect the result of the applied conversion.

Process > Crop…
Interactively specify a 4D (x,y,z,t) region of the data to be displayed in a new viewer window. The same 4D crop will be applied to all channels. The spatial extent of the region can be either specified in voxel units or in physical units (e.g. micrometer).
Motivation: Imaging processes in living samples require setting up imaging parameters before knowing exactly when and where the process of interest takes place. Therefore the imaging field of view (x,y,z) and temporal extent (t) are usually set generously to accommodate sample drift, motion, or growth. Using the crop function one can reduce the dataset to the necessary spatial and temporal dimensions.

Process > Rename…
Rename the data set and channels.

Process > Transform...
Renders an affine view of the data. Motivation: Useful when data is warped due to an acquisition process that renders x-y-z non-orthogonal. Examples are when a stage movement is not orthogonal to the field of view. Also useful in single objective light sheet microscopy (Dunsby, 2008, Bouchard et al. 2015, Sapoznik et al. 2020).

Process > Set Voxel Size…
Changes the voxel size. Motivation: The voxel size may not always be read correctly from the data set, thus it is useful to have the option to set it manually.

Process > Correct Drift
Correct sample motion by interactively creating a 3D track, which will be applied such that the image is stationary relative to the track positions.
Motivation: For time lapse data there is a risk that a sample moves during acquisition. To accommodate for either sample or microscope drift it is common to choose a field of view to encompass expected drift at the expense of larger data footprint. This can be compensated by cropping the data. However, applying a static volumetric crop over the whole time lapse is suboptimal. Therefore an ideal crop would be on drift corrected data (see Supplementary Movie 2). Additional applications can be, e.g., tracking motile cells in tissues.

Process > Correct Drift > Create Track…
Create a 3D track by manually placing anchor points in a subset of time points (track positions in the other time-points will be automatically added by linear interpolation). When done, save the track as a Json file to disk, to be used in [Process > Correct Drift > Apply Track…].

Process > Correct Drift > Apply Track…
Load a 3D track from a file (created with [Process > Correct Drift > Create Track…]) and apply it to the data set. This will cause the dataset to be displayed with each time point shifted according to the track positions (no data duplication).

Supplementary Note 5: Saving

Save > Save as Imaris Volumes…
Save data set as an HDF5 based pyramidal Imaris file (http://open.bitplane.com/ims), with each channel and time point saved as an individual .h5 file and one .ims header file that can be used to view the data both in Fiji's BigDataViewer and in the commercial Imaris software. Motivation: The low data overhead of a pyramidal scheme (in 3D for binning 2 x 2 x 2 at each pyramidal level ca 14%) is a marginal cost for a substantially improved user experience when viewing the data. We therefore provide saving data in an open file format that offers this functionality based on HDF5, which means that it can be handled with all common programming languages.

Save > Save as TIFF Volumes...
Save the dataset as a series of TIFF stacks with each channel and time point saved as an individual .tif file. Motivation: TIFF stacks are still the most used and compatible file format that can be easily opened by all software for downstream analysis.

Save > Save as TIFF Planes...
Save the dataset as a series of TIFF planes, where each z-slice, channel and time point is saved as an individual TIFF file. Motivation: Saving data as a series of TIFF planes is popular in the EM community as already a single volume can be multiple TB in size.

Supplementary Note 6: Misc menu items

Misc > Configure Lazy Loading...
Configure the x, y, and z dimensions of the lazy loading chunks. Motivation: BDP2 lazy loads small chunks from the big image data set, enabling interactive processing of TB sized image data on a standard computer with only a few GB of random access memory. Here, the size of these chunks can be configured. Normally the default values are good and we do not recommend changing them. This menu item has mainly been implemented to facilitate teaching about how different lazy loading schemes affect the performance for different file formats. If the data is loaded via Bio-Formats this setting is currently ignored.

Misc > Show in Hyperstack Viewer
Opens the current image virtually in the "classic" ImageJ hyperstack viewer. Motivation: BigDataViewer is a relatively recent addition to the ImageJ ecosystem and many users are more comfortable using the ImageJ hyperstack viewer. In addition, with the data being displayed in the hyperstack viewer, one has access to many useful ImageJ inspection tools such as intensity histograms and intensity line profiles.

Misc > Configure Logging...
Presents different logging levels, currently: Normal, Debug, and Benchmark. Motivation: For debugging and benchmarking it is very useful to see additional

information, which would however distract in daily routine use. Please be careful using the Benchmark mode, because additional code is executed that may slow down the application.

Supplementary Note 7: Benchmarking

In order to quantify the performance of BDP2 we made a benchmarking scenario consisting of opening a data set, performing a single image processing operation (binning 3x3), browsing single planes and then processing and saving the full dataset, all using a single I/O thread and processing threads using all cores indicated in the hardware configurations. The test dataset had the following characteristics:

- FileType: HDF5
- BitDepth: 16
- nX: 2048, nY: 2048, nZ: 101
- nC: 1 (number of Channels)
- nT: 10 (number of Time points)

A number of different hardware configurations were chosen (for the data storage we use the abbreviation: local area network = LAN).

"**Laptop SSD**": Macbook Pro 2017, 2.5GHz Intel Core i7 (i7-7660U, 4 cores), 16 GB RAM; data storage on onboard SSD.

"**Laptop LAN**": Macbook Pro 2017, 2.5GHz Intel Core i7 (i7-7660U, 4 cores), 16 GB RAM; data access via 1 GBit/s LAN cable.

"**Laptop WIFI**": Macbook Pro 2017, 2.5GHz Intel Core i7 (i7-7660U, 4 cores), 16 GB RAM; data access via 9 MB/s WIFI connection.

"**VM**": Virtual workstation with an Xeon® Processor E5-2698 v3, 2.30 GHz (4 Cores), 32 GB RAM; data access via 10 GBit/s LAN.

"**Cluster**": Computer cluster node with an Intel(R) Xeon(R) Gold 6136 CPU @ 3.00GHz (8 cores), 16 GB RAM; data access via 10 GBit/s LAN.

We evaluated the following performance characteristics:

- Loading (and processing) of a single z-plane
  - Relevant to judge how interactive the application is
- Loading (and processing) of single xyz volume (RPV)
  - Relevant to judge how fast a whole data set could be processed
- Saving of a single xyz volume (SV)
  - Relevant to judge how fast a whole data set could be re-saved

To estimate the total processing and saving (TPS) time of a whole data set with a single I/O thread one can use the following formula:

$$TPS = nC * nT * (RPV + SV) \quad (1)$$

where nC: number of channels, nT: number of timepoints. In a computer cluster environment one can process the different time points in parallel, potentially reducing TPS dramatically.

**Processing Operations**	Laptop SSD	Laptop LAN	Laptop WIFI	Cluster	VM
Read plane (8.4 MB) [s]	0.01	0.15	6	N/A	0.06
Read & process plane [s]	0.1	0.25	6	N/A	0.25
Read volume (847 MB) [s, (MB/s)]	1 (847)	13 (65)	600 (1.4)	2 (423)	2 (423)
Read & process volume [s]	6	18	>600	4	6
Save volume (94 MB) [s]	0.8	2	400	0.5	1.5
Memory usage [GB]	< 4	< 4	< 4	< 4	< 4

Table 1. Benchmarking. Table with data loading, processing and saving times for a typical light sheet microscopy data set (see text for details). In terms of processing operations we performed a 3 x 3 binning in x & y. Binning is a very typical processing step and should be the computationally most expensive of the currently available. Note that this significantly reduces the saving times as, due to the binning, the data to be saved is 9 times smaller than the data to be loaded. All numbers are approximate as, in our hands, even on the same computer the numbers could fluctuate by factors of 3, probably indicating different levels of caching and resource allocations by the underlying operating system. As an output format we chose uncompressed TIFF Stacks, using the ImageJ1 FileSaver.

Supplementary Note 8: Hardware Recommendations

It is recommended that the image data is accessed via a local area network (LAN) cable (e.g., see “Laptop LAN” in Table Benchmarking). For example, accessing the data over a slow (few MB/s) internet connection (e.g., in a home office scenario) can result in update rates of the currently viewed image plane of less than once per second, which is not ideal for interactive browsing of the data (e.g., see “Laptop internet” in Table Benchmarking). For a good user experience tens of MB/s data transfer rate or above is recommended for typical data sets with image planes that are about 2k x 2k pixels in size.

RAM is in general not limiting even for processing of the full data set, because the application tries to only keep the volumes (channels) for one time point in RAM, which typically does not exceed the RAM of a modern laptop (e.g., 16 GB). However, BDP2 offers the option to employ multiple I/O threads (if either the input or output format is HDF5 based we do not recommend using multiple I/O threads as the HDF5 library that we currently use is not capable of multithreading). If multiple I/O threads are chosen, BDP2 processes multiple time points in parallel and the RAM requirements increase linearly as the corresponding data needs to be kept in RAM simultaneously. In practice, finding the optimal number of I/O threads to speed up the processing is hardware and data set dependent and should be tested for each setup.

Regarding the CPU, the processing time will be faster with increasing CPU cores as the processing (e.g. binning) is multi-threaded. In practice, adding more cores may at some point be of limited use, as the overall processing time may become limited by I/O operations (see Table Benchmarking).

Overall, in our experience, the ideal scenario is to use the BDP2 UI to record the processing as a script (currently IJ Macro or Jython) and then execute this script on a computer cluster, parallelising over the time-points to be processed. In order to enable this, we have added a [Record only] button to the saving menu. We already have successfully tested this on a Slurm (Yoo et al. 2003) computer cluster and are happy to consult interested users.

Supplementary Note 9: Lazy-Loading and Lazy-Processing

BDP2 employs Imglib2's `CachedCellImg` class for lazy-loading of image data from files into memory (https://javadoc.scijava.org/ImgLib2/net/imglib2/cache/img/CachedCellImg.html). The `CachedCellImg` partitions an image into small blocks (cells), where a cell's content is read from disk only when the application requests access to this part of the image. Importantly, also all image processing operations are performed lazily, using Imglib2's `Views` (https://javadoc.scijava.org/ImgLib2/net/imglib2/view/Views.html) and `Converter` (https://javadoc.scijava.org/ImgLib2/net/imglib2/converter/Converters.html) classes. To illustrate this, below are two code snippets exemplifying their typical usage in the application.

The first example resembles the **[Process > Convert to 8 bit…]** functionality.

```
RandomAccessibleInterval convertedRai =
     Converters.convert(
           rai,
           new RealUnsignedByteConverter<>( 1000, 3500 ),
           new UnsignedByteType() );
```

The input `rai` (a `RandomAccessibleInterval`) holds the 16-bit (`UnsignedShortType`) image data. The code generates an 8-bit (`UnsignedByteType`) `convertedRai`, mapping values <= 1000 to 0 and >= 3500 to 255. Importantly, even if the input `rai` would represent multiple TB of data, above code would execute instantly, because the conversion is not actually done, but only an instruction of how to convert is wrapped around the input `rai`. Now, if subsequent code would request an actual pixel value of the `convertedRai`

```
value = convertedRai.randomAccess().setPositionAndGet(
100,100,50,4,3 ).getRealDouble();
```

only the minimally required computations to compute this one pixel value at the specific location `(100,100,50,4,3)` (x,y,z,c,t) are executed, without converting any other values.
See
https://github.com/bigdataprocessor/bigdataprocessor2/blob/0.5.6/src/main/java/de/embl/cba/bdp2/process/convert/MultiChannelUnsignedByteTypeConverter.java for a snapshot of the actual code in the application.

The second example resembles the **[Process > Align channels...]** functionality.

```
channelRai = Views.hyperSlice( rai, DimensionOrder.C,
channelIndex );
shiftedChannelRai = Views.translate( channelRai, new
long[]{10,0,0} );
```

The first line extracts part of the 5D input `rai`, namely only one of the channels. The second line shifts this channel by 10 pixels along the x-axis (potentially correcting microscope misalignments during the acquisition). Again, these lines of code execute instantly even for a TB sized data set, because they only attach instructions for how to access the input data without actually accessing or copying any data. If now subsequent code would access the `shiftedChannelRai`

```
value =
shiftedChannelRai.randomAccess().setPositionAndGet(100,100,50,
3).getRealDouble();
```

only the value for a single pixel would be fetched from the input `rai`, taking into account the shift of 10 pixels and the channel subsetting, without touching any of the other pixels.
See
https://github.com/bigdataprocessor/bigdataprocessor2/blob/0.5.6/src/main/java/de/embl/cba/bdp2/process/align/channelshift/ChannelShifter.java for a snapshot of the full code in the application.

Supplementary Movie 1: BigDataProcessor2 Workflow

https://www.youtube.com/embed/OixZ0ILbkvc?vq=hd1440

Opening, browsing and preprocessing of a 250GB HDF5 based raw data set. Movie shows a screen recording of a basic preprocessing workflow of a 250 GB HDF5 image data set acquired by light-sheet microscopy. The following steps are demonstrated: Open Luxendo HDF5 -> Brightness & Color adjustment -> Set Voxel Size -> Align Channels -> Crop -> Bin -> Save. The 2 color early mouse embryo data were provided by Manuel Eguren, Ellenberg group EMBL Heidelberg.

Supplementary Movie 2: Drift Correction

https://www.youtube.com/embed/7SCZIToxY9E?vq=hd1440

Drift correction of a 250 GB HDF5 data set. The movie shows a screen recording of the drift correction of a 250 GB HDF5 image data set acquired by light-sheet microscopy. The following steps are shown in the movie: Correct Drift -> Create Track -> Apply Track. The single color mouse mammary gland organoid data were provided by Ashna Alladin, Jechlinger group, EMBL Heidelberg.

Supplementary References:

Dunsby C. (2008). Optically sectioned imaging by oblique plane microscopy. *Optics Express*. **16**, 20306-20316.

Bouchard M., Voleti V., Mendes C., Lacefield C., Grueber W., Mann R., Bruno R. and Hillman C. (2015). Swept confocally-aligned planar excitation (SCAPE) microscopy for high-speed volumetric imaging of behaving organisms. *Nature Photonics.* **9**, pages 113–119.

Juneau J., Baker J., Wierzbicki F., Muoz L., Ng V., Ng A. and Baker D. (2010). The Definitive Guide to Jython: Python for the Java Platform ISBN-13 (pbk): 978-1-4302-2527-0

Linkert M., Rueden C., Allan C., Burel J.-M., Moore W., Patterson A., Loranger B., Moore J., Neves C., MacDonald D., Tarkowska A., Sticco C., Hill E., Rossner M., Eliceiri K. and Swedlow J. (2010). Metadata matters: access to image data in the real world. *J. Cell Biol.* 189 (5): 777–782. https://doi.org/10.1083/jcb.201004104

Sapoznik E., Chang B-J, Huh J., Ju R., Azarova E., Pohlkamp T., Welf S., Broadbent D., Carisey A., Stehbens S., Lee K-M., Marín A., Hanker A., Schmidt J., Arteaga C.,Yang B., Kobayashi Y., RaoTata P., Kruithoff R., Doubrovinski K., Shepherd D., Millett-Sikking A., York A., Dean K. and Fiolka R. (2020) A versatile oblique plane microscope for

large-scale and high-resolution imaging of subcellular dynamics. eLife 2020;9:e57681 DOI: 10.7554/eLife.57681.

Sarkans U., Gostev M., Athar A., Behrangi E., Melnichuk O., Ali A., Minguet J., Rada J., Snow C., Tikhonov A., Brazma A. and McEntyre J. (2017). The BioStudies database—one stop shop for all data supporting a life sciences study, *Nucleic Acids Research*, **6** 1266-1270.

Schneider C., Rasband W. and Eliceiri K. (2012) NIH Image to ImageJ: 25 years of image analysis. *Nat. Methods*, **9**, 671–675.

Yoo A., Jette M. and Grondona M., Job Scheduling. Slurm: Simple Linux Utility for Resource Management, Strategies for Parallel Processing, volume 2862 of Lecture Notes in Computer Science, pages 44-60, Springer-Verlag, 2003.
